# Complete Genome Sequence of *Bradyrhizobium* sp. Strain BDV5040, Representative of Widespread Genospecies B in Australia

**DOI:** 10.1128/MRA.01326-20

**Published:** 2021-01-21

**Authors:** Christine Oger-Desfeux, Jérôme Briolay, Philippe M. Oger, Bénédicte Lafay

**Affiliations:** a Université de Lyon, Université Claude Bernard Lyon 1, PRABI-ASMB, FR3728 Bio-Environnement et Santé, Villeurbanne, France; b Université de Lyon, Université Claude Bernard Lyon 1, CNRS, DTAMB, FR3728 Bio-Environnement et Santé, Villeurbanne, France; c Université de Lyon, INSA de Lyon, CNRS, UMR5240, Villeurbanne, France; d Université de Lyon, École Centrale de Lyon, CNRS, UMR5005, Laboratoire Ampère, Écully, France; e Université de Lyon, Université Claude Bernard Lyon 1, CNRS, UMR5558, Laboratoire de Biométrie et Biologie Évolutive, Villeurbanne, France; University of Arizona

## Abstract

We report the complete genome sequence of *Bradyrhizobium* sp. strain BDV5040, representative of *Bradyrhizobium* genospecies B, which symbiotically associates with legume hosts belonging to all three Fabaceae subfamilies across the Australian continent. The complete genome sequence provides a genetic reference for this Australian genospecies.

## ANNOUNCEMENT

*Bradyrhizobium* sp. strain BDV5040 was isolated in 1995 from a root nodule of *Bossiaea ensata* (Fabaceae, Faboideae, Bossiaeeae) collected in Ben Boyd National Park, New South Wales, Australia (37°12′S, 149°57′E; altitude, 140 m), in the course of a survey of rhizobia associated with native shrubby legumes in southeastern Australia (1). It is a representative of *Bradyrhizobium* genospecies B, which occurs under different climatic and edaphic conditions across the whole Australian continent and exhibits a broad host range encompassing all three Fabaceae subfamilies (1–4).

Strain BDV5040 was grown from a lyophilized stock in 30 ml of yeast extract mannitol broth (5) at 25°C and 200 rpm for 5 days. Genomic DNA was prepared by successive phenol-chloroform extractions as described (6). DNA quantification and quality control were performed using a NanoDrop spectrophotometer, a Qubit 4 fluorometer, and agarose gel electrophoresis. The same DNA was used for Nanopore and Illumina sequencing. Illumina libraries were obtained using the Nextera XT kit following the manufacturer’s instructions, starting with 1 ng of genomic DNA, and were analyzed by paired-end 2 × 300-bp sequencing on a MiSeq instrument. Poor-quality regions (Q < 30) of raw reads were removed using Sickle v1.33 (7). Long reads were obtained with the Oxford Nanopore Technologies MinION FLO-MIN106 flow cell from a library prepared with the SQK-RAD004 kit using 1 μg of DNA and 20-second tagmentation. Base calling was performed using Guppy v3.1.5. Sequence quality was assessed using FastQC v0.11.9 (8) for Illumina reads and MinIONQC.R v1.4.2 (9) for Nanopore reads. The preprocessing of Illumina sequencing data using Trimmomatic v0.39 (10) (parameters: ILLUMINACLIP:TruSeq3-PE-2.fa:2:30:10:2:keepBothReads LEADING:3 TRAILING:3 SLIDINGWINDOW:4:15 MINLEN:50) resulted in 3.87 million paired-end reads (mean lengths of 222 bp and 160 bp for forward and reverse reads, respectively, with ∼150× coverage). Nanopore long reads (total of 2.7 Gbp, with an *N*_50_ value of 26,211 bp) were filtered and trimmed for length (>1,500 bp) and quality (score of >8) using Nanofilt v2.5.0 (11), and adapters were removed using Porechop v0.2.4 (12). Long reads were further reduced to 800 Mbp as a target quantity using Filtlong v0.2.0 (13) (parameters: --min_length 2000 --keep_percent 90 --target_bases 800000000). Illumina and Nanopore reads were coassembled using Unicycler v0.4.8 (14) with default parameters, resulting in a single component with eight segments and incomplete status (length, 7,622,333 bp; *N*_50_, 7,339,313 bp). Completion was obtained by exporting the sequence path from Bandage v0.8.1 (15) and filling a last gap using Pilon v1.23 (16) and by manually comparing the sequence with Unicycler 003_long_read_assembly.fasta. The assembly and complete chromosome sequence were carefully inspected by visualizing the alignment of long and short reads using minimap2 v2.17 (17) and IGV v2.7.2 (18). Finally, the chromosome was rotated to start at *dnaA*.

The circular chromosome is 7,622,528 bp long, with an average G+C content of 63.92%. The sequence was automatically annotated by the NCBI Prokaryote Genome Annotation Pipeline (PGAP) v4.13 (19). The genome consists of 7,092 protein-coding genes, 48 tRNAs, 1 copy each of the 5S, 16S, and 23S rRNA genes, and 88 pseudogenes.

### Data availability.

The genome sequence of *Bradyrhizobium* genospecies B strain BDV5040 is available in NCBI GenBank under accession number CP061379. The raw sequence reads are available under SRA accession numbers SRX9514896 and SRX9514898 under BioProject number PRJNA662585 and BioSample number SAMN16089659.
